# Rising Klebsiella pneumoniae Infections and Its Expanding Drug Resistance in the Intensive Care Unit of a Tertiary Healthcare Hospital, Saudi Arabia

**DOI:** 10.7759/cureus.10060

**Published:** 2020-08-26

**Authors:** Ali Al Bshabshe, Ahmed Al-Hakami, Basel Alshehri, Khalid A Al-Shahrani, Abdullah A Alshehri, Mohammed B Al Shahrani, Ibrahim Assiry, Martin R Joseph, Abdullah Alkahtani, Mohamed E Hamid

**Affiliations:** 1 Department of Medicine, King Khalid University, Abha, SAU; 2 Department of Clinical Microbiology and Parasitology, King Khalid University, Abha, SAU; 3 Internal Medicine, King Khalid University, Abha, SAU; 4 Intensive Care Unit, Aseer Central Hospital, Abha, SAU

**Keywords:** nosocomial infections, antibiogram, carbapenem-resistant, aseer region

## Abstract

Nosocomial infections caused by *Klebsiella pneumoniae* and other Gram-negative organisms have emerged as a significant health problem especially in intensive care units (ICU). This study aims to examine *K. pneumoniae* infections in the ICU of Aseer Central Hospital and to determine their antimicrobial susceptibility and their relationship to patients' clinical outcomes. This is a retrospective observational study done in a tertiary care center in the Aseer region in Saudi Arabia. The study spanned from January 2018 to December 2019. Demographic, microbiologic, and patient outcomes were collected from 276 patients with various infections. Identification of isolates and in vitro susceptibility to 32 antimicrobial agents were done by the Vitek 2 automated system (bioMérieux, Marcy-l'Étoile, France). Prevalence of *K. pneumoniae* bacteria, their susceptibility to antimicrobials, and effect on clinical outcome were studied. Two hundred seventy-six *K. pneumoniae* were recovered from ICU patients with various infections. *K. pneumoniae* isolates (n=276) were collected mainly from the respiratory tract (61%) and *K. pneumoniae* represented 39% of the major causal agents of ICU infections, followed by *Acinetobacter* spp. (30%), *Pseudomonas aeruginosa* (10.0%), *Escherichia coli* (7%), and others (14%). The mortality among the 276 ICU patients was 33.3%; *K. pneumoniae* was connected to 42% of the cases and 67% of the total deaths were between 50 and 90 years of age. *K. pneumoniae* demonstrated high sensitivity and hence can be recommended for in vivo treatment for tigecycline (81%), cefazolin (77.2%), colistin (64.9%), and to a lesser extent norfloxacin (60%) and imipenem (55.5%). High resistance was detected for ampicillin (100%), extended-spectrum β-lactamases-sulbactam (ESBL-SCM) (100%), piperacillin (100%), and ceftazidime (92.5%). Resistance to carbapenems was elevated in ertapenem (65.2%) and meropenem (61.7%). The increase of *K. pneumoniae* represents a threat to ICU patients, although *K. pneumoniae* infections were results rather than the causes, as it was connected to almost half of the ICU mortalities. Tigecycline alone or in combination with colistin on high-dose regimens could be a more effective therapy for treating carbapenem-resistant *K. pneumoniae* infections.

## Introduction

In the last two decades, *Klebsiella ​​*​*pneumoniae *has emerged as a clinically important bacteria because of increased antibiotic resistance and the tendency to acquire antibiotic resistance and to initiate serious outcomes [1,2]. In recent years, *K. pneumoniae *has been identified as a major cause of hospital-acquired pneumonia and is responsible for approximately 10% of all hospital-acquired infections, ranking second among Gram-negative pathogens [3].

The occurrence of multidrug-resistant organisms (MDR) in intensive care units (ICU) is rising globally with variances among countries, causal agents, and hospital settings [4]. The rapid emergence of MDR Gram-negative bacteria and fungi is currently causing significant healthcare risks that need solid prevention and control plans. Globally novel epidemiologic arrangements of ICU occurrence have been watched for Gram-positive multidrug-resistant organisms [5,6]. Healthcare-acquired infections, also known as nosocomial infections, caused by *K. pneumoniae*, *Acinetobacter baumannii*, and other Gram-negative organisms are serious emerging problems worldwide. Continued surveillance and finding a suitable drug is of central priority [7]. Drug-resistant *Enterobacteriaceae*, particularly *Klebsiella *spp., have emerged as a major health problem globally [8]. Long-term acute care hospitals have a unique patient population, with multiple risk factors for carbapenem-resistant *Enterobacteriaceae *colonization and infection [9]. Resistance among *K. pneumoniae *in particular to the last-resort antibiotics carbapenems and colistin is mounting globally [10]. Methicillin-Resistant *Staphylococcus aureus* (MRSA), carbapenem-resistant *P. aeruginosa*, and extended-spectrum β-lactamases (ESBL)-producing *K. pneumoniae *are of major concern worldwide. Good hand hygiene and rigorous aseptic practices remain the best practice for infection control and consistent methods and definitions of compliance monitoring are essential to compare results throughout various settings [11].

Surveillance of antimicrobial resistance is vital to guiding the empirical treatment of infections. Ordering and reporting routine data on clinical isolate testing may offer more timely information about resistance patterns than traditional surveillance network methods. Studies have demonstrated that the applicability of routine surveillance guidelines is important in empirical antimicrobial therapy and essential patient care assuming the continuous evolution of bacterial susceptibility. It is essential to know the significance of antimicrobial stewardship, to be acquainted with when to refer to infectious disease specialists for leadership, and to be able to distinguish situations when antimicrobial therapy is not required [12]. Antibiotic stewardship programs have been associated with reductions in antimicrobial use, duration of stay, and costs with no negative impact on mortality and should be widely promoted in ICUs [5].

Carbapenem-resistant *Enterobacteriaceae *represents an urgent threat worldwide. Carbapenem-resistant *K. pneumoniae *is emerging as a risk to public health as it causes various nosocomial infections, notably respiratory tract infections. Patients in the ICU are at a bigger risk of hospital-acquired *K. pneumoniae *infection, particularly infections caused by carbapenem-resistant *K. pneumoniae* [13]. Carbapenems, a β-lactam antibiotic, target cells by inhibiting transpeptidases which prevent the synthesis of peptidoglycan, a necessary structural component, leading to cell lysis. A study that investigated 49 papers found that the rates of resistance to carbapenem in *K. pneumoniae *and *E. coli *were 24.0% (95% confidence interval [CI] 18.0-31.0) and 5.0% (95% CI 2.0-8.0), respectively. The blaOXA-48 gene was the most common cause of carbapenem resistance in *K. pneumoniae *and *E. coli *[14].

Hospital-acquired bacterial infections pose a tough challenge for healthcare providers because patients initially demand empirical treatment since the delay of appropriate initial antimicrobial therapy increases morbidity and mortality among affected patients [15]. Appropriate microbiologic surveillance and estimate of antimicrobial resistance are vital for confronting nosocomial infections [16].

The present study was planned to investigate* K. pneumoniae* infections in the ICU of Aseer Central Hospital, determine their antimicrobial sensitivities, and their association to patents' clinical outcomes.

## Materials and methods

The present investigation was a retrospective observational study based on data collected from patients in the ICU in Aseer Central Hospital, a tertiary care hospital, in the southern region of Saudi Arabia, from 2018-2019. The study group comprised patients admitted for more than 48 h in the ICU and who had a hospital-acquired infection. Data were collected regarding demographic, microbiologic, antimicrobial treatment, and patient outcomes. In this analysis, a total of 276 patients (112 patients in 2018, and 164 patients in 2019) were classified as healthcare-acquired or nosocomial infections (those occurring within 48 h of hospital admission), with bacteremia, pneumonia, and other infections.

Procedures performed in this research affecting human participants and sampling were per the ethical standards. Ethical approval was obtained from Aseer Central Hospital Ethics, Internal Review Board Committee (ACH IRB No. 20191203).

Clinical specimens from different infections in the ICU (n=276) were processed for culture by conventional methods. Initial identification was performed using standard phenotypic tests [17]. Subsequent initial identification, confirmation of isolates was done at the microbiology laboratory using the Vitek 2 identification system (BioMerieux, Marcy-l'Étoile, France) according to manufacture criteria.

Antimicrobial susceptibility testing was also carried out using the Vitek 2 system, according to the manufacturer's information. The in vitro susceptibility of main antimicrobial agents (n=32) to leading ICU pathogens (2018-2019). The 32 antimicrobial agents were tested for at least 10 or more strains; other agents were excluded.

The Statistical Product and Service Solutions (SPSS), version 25 (IBM Corp., Armonk, NY) was used for the analysis. Descriptive analysis including frequency and percent distribution was performed for all variables including bio-demographic data, source of clinical specimens, and type of organisms. Univariate analysis was used to assess the crude relation between different studied factors and ICU patient outcomes (death, discharged) using the crude odds ratio.

## Results

The description of patient baseline characteristics of ICU patients in Aseer Central Hospital (2018-2019) is shown in Table 1. Of the total 276 patients 83 were females (30%) and 193 were males (70%) in two years: 2018 (n=112) and 2019 (n=164) with different age groups. The organism was recovered from different clinical sources but mainly the respiratory tract (169; 61.0%), blood (38; 14.0%), urine samples (36; 13%), wound and skin swab (21; 8.0%), and other (12; 4.3%).

**Table 1:** Description of patient baseline characteristics obtained from the ICU, Aseer Central Hospital (2018-2019).

Criteria		Died		Discharged		Total		Chi square	Sig.
		N	%	N	%	N	%		
Year	2018	41	45	71	39	112	41	0.91	0.34
	2019	51	55	113	61	164	59		
Gender	Female	48	52	35	19	83	30	31.12	0.00
	Male	44	48	149	81	193	70		
					0				
Age	<10 yr	1	1	2	1	3	1	32.05	0.00
	10-19 yr	3	3	22	12	25	9		
	100-109 yr	1	1	0	0	1	0		
	20-29 yr	3	3	43	23	46	17		
	30-39 yr	8	9	29	16	37	13		
	40-49 yr	10	11	17	9	27	10		
	50-59 yr	9	10	13	7	22	8		
	60-69 yr	12	13	18	10	30	11		
	70-79 yr	24	26	21	11	45	16		
	80-89 yr	17	18	15	8	32	12		
	90-99 yr	4	4	4	2	8	3		
Specimen	Abdominal Aspiration	0	0	2	1	2	1	1.17	0.28
	Abdominal fluid and aspirate	0	0	1	1	1	0		
	Blood	13	14	25	14	38	14		
	CSF	4	4	0	0	4	1		
	Pus, discharge and swab	2	2	3	2	5	2		
	Respiratory specimen	61	66	108	59	169	61		
	Urine	8	9	28	15	36	13		
	Wound and skin swab	4	4	17	9	21	8		
					0				
Organism	Acinetobacter baumannii	10	11	14	8	24	9	1.02	0.31
	Acinetobacter baumannii – haemolyticus	22	24	36	20	58	21		
	Citrobacter sp.	0	0	1	1	1	0		
	coagulase - negative Staphylococcus spp.	0	0	1	1	1	0		
	Enterobacter aerogenes	0	0	1	1	1	0		
	Enterobacter cloacae	2	2	6	3	8	3		
	enterococcus faecium	0	0	1	1	1	0		
	Escherichia coli	3	3	16	9	19	7		
	Klebsiella oxytoca	1	1	2	1	3	1		
	Klebsiella pneumoniae	39	42	68	37	107	39		
	Morganella morganii	1	1	0	0	1	0		
	Proteus mirabilis	2	2	4	2	6	2		
	Providencia stuartii	1	1	2	1	3	1		
	Pseudomonas aeruginosa	7	8	21	11	28	10		
	Pseudomonas sp.	0	0	1	1	1	0		
	Serratia marcescens	1	1	2	1	3	1		
	Staphylococcus aureus	2	2	8	4	10	4		
	Staphylococcus epidermidis	1	1	0	0	1	0		

**Figure 1:**
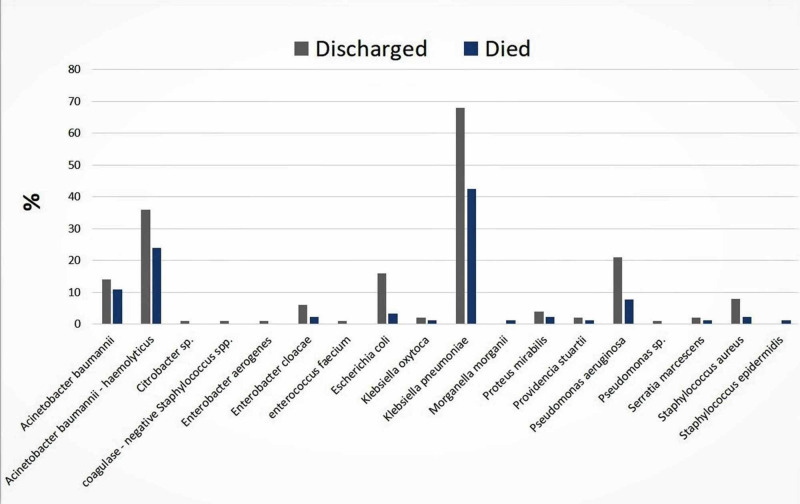
Prevalence of Klebsiella pneumoniae in relation to other causal agents in the ICU infections and their counts according to patient outcomes (2018-2019).

*K. pneumoniae* was the dominant isolate (39.0%), followed by *Acinetobacter *spp. (30.0%), *E. coli* (10.7%), *Pseudomonas aeruginosa* (10.7%), *Staphylococcus aureus* (7.3%), *Enterobacter cloacae* (2.7%), *Proteus mirabilis* (1.3%), *Citrobacter* sp. (0.7%), and *Serratia marcescens* (0.7%). The death rate among the 276 patients was 36.0%, of which *K. pneumoniae* was linked to 42% and *Acinetobacter* spp. were associated with 35% of the cases (Figure 1).

The in vitro susceptibility of *K. pneumoniae* to main antimicrobial agents is shown in Table 2. *K. pneumoniae* was found variable in susceptibility to the tested antimicrobial agents. The antimicrobials with high sensitivity and hence can be recommended for the in vivo treatment were: tigecycline (81.0%), cefazolin (77.2%), norfloxacin (60.0%), colistin (64.9%), and to a lesser extent imipenem (55.5%). On the other hand, high rates of resistance were noticed in ampicillin, extended-spectrum β-lactamases-sulbactam (ESBL-SCM), piperacillin (100%), and to a lesser extent ceftazidime (92.5%), minocycline (80.2%), ceftriaxone (80.1%), and tetracycline (80%).

**Table 2:** In vitro susceptibility of Klebsiella pneumoniae to common antimicrobial agents. ESBL-SCM: extended-spectrum β-lactamases-sulbactam

Antimicrobial agents	% Sensitive	% Resistant
Amikacin	44.7%	55.3%
Amoxicillin/ K Clavulanate	32.1%	67.9%
Ampicillin-sulbactam	20.8%	79.2%
Ampicillin	0.0%	100.0%
Aztreonam	20.9%	79.1%
Cefazolin	77.2%	22.8%
Cefepime	21.3%	78.7%
Cefitriaxone	17.9%	82.1%
Cefotaxime	31.1%	68.9%
Cefoxitin	37.2%	62.8%
Ceftazidime	32.7%	67.3%
Ceftazidime	7.5%	92.5%
Cefuroxime	27.5%	72.5%
Ciprofloxacin	35.8%	64.2%
Colistin	64.9%	35.1%
Ertapenem	34.8%	65.2%
ESBL- SCM	0.0%	100.0%
Gentamicin	42.9%	57.1%
Imipenem	55.5%	44.5%
Levofloxacin	36.8%	63.2%
Meropenem	38.3%	61.7%
Minocycline	19.8%	80.2%
Moxifloxacin	31.1%	68.9%
Nitrofurantoin	25.0%	75.0%
Norfloxacin	60.0%	40.0%
Piperacillin/ tazobactam	32.4%	67.6%
Piperacillin	0.0%	100.0%
Tetracycline	20.0%	80.0%
Ticarcillin/ K Clavulanate	50.0%	50.0%
Tigecycline	81.0%	19.0%
Tobramycin	29.7%	70.3%
Trimethoprim/ sulfamethoxazole	38.9%	61.1%

**Figure 2:**
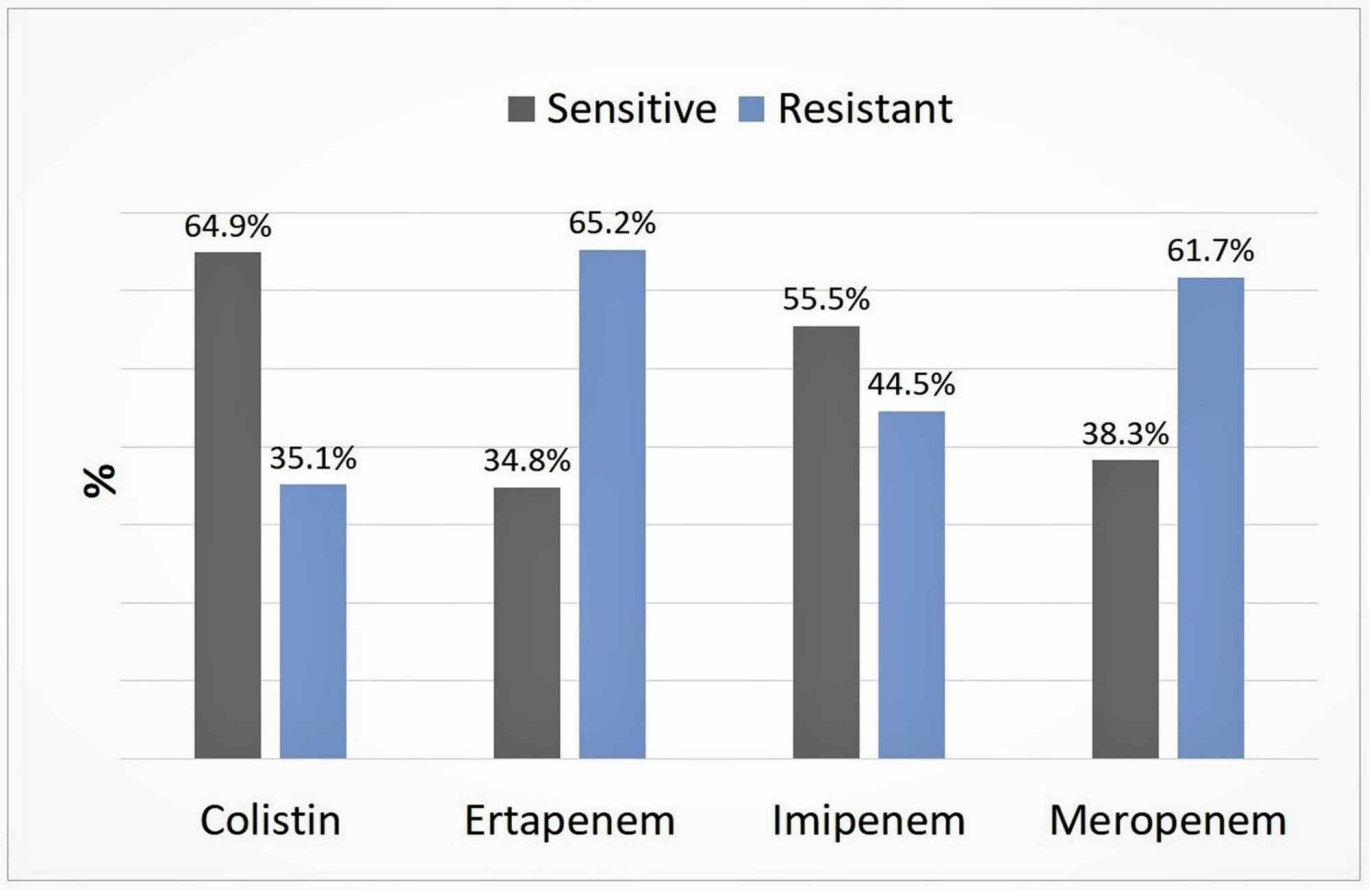
Susceptibility of Klebsiella pneumoniae recovered from patients in ICU of Aseer Central Hospital to carbapenems.

The susceptibility of *K. pneumoniae* to carbapenems is shown in Figure 2. The resistance rate to ertapenem and meropenem was high (65.2% and 61.7%, respectively). On the other hand, colistin and imipenem were relatively lower (35.1% and 44.5%, respectively).

## Discussion

The present study determined that *K. pneumoniae* is a major ICU pathogen and possesses the ability to evolve and broaden its resistance to many antimicrobial agents. We presented this data to enhance awareness amongst emergency physicians and intensivists of the early diagnosis and antimicrobial assay to evade complications including death. *K. pneumoniae* has a wide habitat both in nature and in hospital settings and considered one of the most opportunistic pathogens that causes many infections in humans, for instance pneumonia and other respiratory tract infections, bloodstream infections, urinary tract infections, and surgical-site infections [18].

It is not fully known how *K. pneumoniae* can cause disease. Clonal analyses of *K. pneumoniae* strains showed that there are distinctive clonal groups, some of which may be associated with specific *K. pneumoniae* disease syndromes [19]. Several investigations from single medical centers or individual countries have reported mortality rates in patients infected with carbapenem-resistant *K. pneumoniae*. Researchers have shown conflicting results regarding mortality rates among persons infected with *K. pneumoniae* isolates as some reported high mortalities while others reported contrary results [20]. Our results supported the high mortality since we have registered a 50% mortality among *K. pneumoniae*-infected patients. A meta-analysis by Falagas et al. [18] reported higher all-cause mortality among patients infected with carbapenem-resistant *Enterobacteriaceae* than in those with carbapenem-susceptible infections.

It is not fully understood how nosocomial infections are caused by *K. pneumoniae*. The possible reasons for the spread of nosocomial infections in general and those caused by *K. pneumoniae*, in particular, is related to the health setting and its hygienic measures [21] and secondly due to the ability of *K. pneumoniae* to acquire resistant genes via horizontal gene transfer facilitated by plasmids and mobile genetic elements [22]. 

Carbapenem-resistant *K. pneumoniae* is an emerging multi-drug resistant healthcare-associated pathogen. It is connected to a high-level of mortality and deprived outcomes irrespective of the treatment course of therapy using tigecycline or other selected therapy [23]. Our results are in line with this study; we noticed a high level of resistance among our isolates amounting to 61.7% in the case of meropenem, 44.5% in imipenem, and 35.1% in colistin.

As practiced in many health care settings [24], tigecycline is used for the treatment of carbapenem-resistant *Enterobacteriaceae* infections, including *K. pneumoniae*. Tigecycline was found the best choice from the in vitro results reported in the present study as the susceptibility of *K. pneumoniae* was 81.9%. Our results indicated that the efficacy of tigecycline in inhibiting *K. pneumoniae* in vitro is similar to that of other antibiotics. Tigecycline combination therapy and high-dose regimens may be more effective than monotherapy and standard-dose regimens, respectively. Nonetheless, considering that the currently available evidence is limited, well-designed randomized controlled trials are urgently needed to clarify the comparative efficacy of tigecycline in treating carbapenem-resistant *Enterobacteriaceae* (CRE) infections [25]. Efficacy of tigecycline-colistin combination in the treatment of carbapenem-resistant *K. pneumoniae* endocarditis. The patient was treated with the colistin-tigecycline combination, with a favorable outcome. In conclusion, the colistin-tigecycline combination may be a possible combination in the therapy of infective endocarditis caused by MDR *Enterobacteriaceae* [26].

In the present study, 42% of the ICU mortalities were linked to *K. pneumoniae* infections and these infections were mainly derived from a respiratory origin (61%). A previous study indicated that unusual carriage of multi-resistant *K. pneumoniae* and/or *Acinetobacter baumannii* was observed among severely sick patients which had predisposed them to secondary nosocomial infections that significantly affected the mortality [27].

No molecular work was done to detect the reasons behind such resistance. Carbapenem-resistant strains have increased rapidly, rising from 1.6 to 10.4% associated with central line blood-stream infections between 2001 and 2011 in the United States, and have aroused widespread attention, presenting a challenge because the antimicrobial treatment options remain very restricted. A high percentage of ESBL-producers among clinical isolates of *K. pneumoniae* and a high rate of multidrug resistance was reported from the Armed Forces Hospital, Al-Kharaj, Saudi Arabia, from November 2004 to October 2007. This study urged for continued infection control measures and prudent use of antimicrobial agents is essential in reducing the spread of multi-resistant ESBL-producing *K. pneumoniae* [28]. *K. pneumoniae* has recently gained notoriety as an infectious agent due to a rise in the number of severe infections and the increasing scarcity of effective treatments. These concerning circumstances have arisen due to the emergence of *K. pneumoniae* strains that have acquired additional genetic traits and become either hypervirulent (HV) or antibiotic-resistant. Much work is left to be done in characterizing these newly discovered factors, understanding how infections differ between healthy and immunocompromised patients and identifying attractive bacterial or host targets for treating these infections [29].

Intensive care units (ICUs) are high-risk areas for transmission of antimicrobial-resistant bacteria, but no controlled study has tested the effect of rapid screening and isolation of carriers on transmission in settings with best-standard precautions. We assessed the frequency of common causal organisms and their antimicrobial susceptibility to check upon the treatment strategies and their effect on health outcomes.

## Conclusions

The present study concluded that *K. pneumoniae* is a major ICU pathogen and possesses the ability to evolve and broaden its resistance to many antimicrobial agents. Fifty percent of ICU mortalities were caused by *K. pneumoniae* and infections were mainly derived from the respiratory origin (60%). The occurrence of *K. pneumoniae* and its antimicrobial resistance were rising from our two-year comparison. These increases were also noticed among other Gram-negative bacteria, notably *Acinetobacter* spp. and *Pseudomonas aeruginosa*.

This current situation is alarming and requires great attention from health authorities to decrease nosocomial infections, in particular in ICU settings. The potential of hospital personnel to spread nosocomial pathogens from person to person is very serious, it is recommended that hospitals should implement Centers for Disease Control and Prevention proposals to decrease nosocomial bloodstream infections. *K. pneumoniae* infections were on the increase as checked in 2018 and 2019 and becoming a threat to ICU patients. They were associated with half of the ICU cohort in the study samples and may well be a contributing factor to mortality. Tigecycline, colistin, and imipenem can be recommended as first-line treatment of *K. pneumoniae* infections. The study recommends the application of routine in vitro assays to assure prompt treatment to avoid complications to already vulnerable ICU patients from the rising drug-resistant bacteria, notably *K. pneumoniae*.

## References

[1] Nordmann P, Cuzon G, Naas T (2009). The real threat of Klebsiella pneumoniae carbapenemase-producing bacteria. Lancet.

[2] Lima AM, de Melo ME, Alves LC (2014). Investigation of class 1 integrons in Klebsiella pneumoniae clinical and microbiota isolates belonging to different phylogenetic groups in Recife, State of Pernambuco. Rev Soc Bras Med Trop.

[3] Abdelsalam MF, Abdalla MS, El-Abhar HS (2018). Prospective, comparative clinical study between high-dose colistin monotherapy and colistin-meropenem combination therapy for treatment of hospital-acquired pneumonia and ventilator-associated pneumonia caused by multidrug-resistant Klebsiella pneumoniae. J Glob Antimicrob Resist.

[4] Teerawattanapong N, Panich P, Kulpokin D (2018). A systematic review of the burden of multidrug-resistant healthcare-associated infections among intensive care unit patients in southeast Asia: the rise of multidrug-resistant Acinetobacter baumannii. Infect Control Hosp Epidemiol.

[5] Kerneis S, Lucet JC (2019). Controlling the diffusion of multidrug-resistant organisms in intensive care units. Semin Respir Crit Care Med.

[6] McGowan JE, Jr Jr (2001). Increasing threat of Gram-positive bacterial infections in the intensive care unit setting. Crit Care Med.

[7] Vakili B, Fazeli H, Shoaei P (2014). Detection of colistin sensitivity in clinical isolates of Acinetobacter baumannii in Iran. J Res Med Sci.

[8] Hussein K, Raz-Pasteur A, Finkelstein R (2013). Impact of carbapenem resistance on the outcome of patients' hospital-acquired bacteraemia caused by Klebsiella pneumoniae. J Hosp Infect.

[9] Igbinosa O, Dogho P, Osadiaye N (2020). Carbapenem-resistant Enterobacteriaceae: a retrospective review of treatment and outcomes in a long-term acute care hospital. Am J Infect Control.

[10] Berglund B, Hoang NTB, Tarnberg M (2018). Insertion sequence transpositions and point mutations in mgrB causing colistin resistance in a clinical strain of carbapenem-resistant Klebsiella pneumoniae from Vietnam. Int J Antimicrob Agents.

[11] Carter EJ, Pouch SM, Larson EL (2014). Common infection control practices in the emergency department: a literature review. Am J Infect Control.

[12] Leekha S, Terrell CL, Edson RS (2011). General principles of antimicrobial therapy. Mayo Clin Proc.

[13] Qin X, Wu S, Hao M (2020). The colonization of carbapenem-resistant Klebsiella pneumoniae: epidemiology, resistance mechanisms, and risk factors in patients admitted to intensive care units in China. J Infect Dis.

[14] Nasiri MJ, Mirsaeidi M, Mousavi SMJ (2020). Prevalence and mechanisms of carbapenem resistance in Klebsiella pneumoniae and Escherichia coli: a systematic review and meta-analysis of cross-sectional studies from Iran. Microb Drug Resist.

[15] Lode H (2005). Management of serious nosocomial bacterial infections: do current therapeutic options meet the need?. Clin Microbiol Infect.

[16] Lee CY, Chen PY, Huang FL (2009). Microbiologic spectrum and susceptibility pattern of clinical isolates from the pediatric intensive care unit in a single medical center - 6 years' experience. J Microbiol Immunol Infect.

[17] Murray PR, Baron EJ: (2007). Manual of Clinical Microbiology. 9th ed.

[18] Falagas ME, Tansarli GS, Karageorgopoulos DE (2014). Deaths attributable to carbapenem-resistant Enterobacteriaceae infections. Emerg Infect Dis.

[19] Broberg CA, Palacios M, Miller VL (2014). Klebsiella: a long way to go towards understanding this enigmatic jet-setter. F1000prime reports.

[20] Xu L, Sun X, Ma X (2017). Systematic review and meta-analysis of mortality of patients infected with carbapenem-resistant Klebsiella pneumoniae. Ann Clin Microbiol Antimicrob.

[21] Schmidt JS, Kuster SP, Nigg A (2020). Poor infection prevention and control standards are associated with environmental contamination with carbapenemase-producing Enterobacterales and other multidrug-resistant bacteria in Swiss companion animal clinics. Antimicrob Resist Infect Control.

[22] Hao M, Shen Z, Ye M (2019). Outbreak Of Klebsiella pneumoniae carbapenemase-producing Klebsiella aerogenes strains in a tertiary hospital In China. Infect Drug Resist.

[23] Neuner EA, Yeh JY, Hall GS (2011). Treatment and outcomes in carbapenem-resistant Klebsiella pneumoniae bloodstream infections. Diagn Microbiol Infect Dis.

[24] Chen F, Shen C, Pang X (2020). Effectiveness of tigecycline in the treatment of infections caused by carbapenem-resistant gram-negative bacteria in pediatric liver transplant recipients: a retrospective study. Transpl Infect Dis.

[25] Wunderink RG, Giamarellos-Bourboulis EJ, Rahav G (2018). Effect and safety of meropenem-vaborbactam versus best-available therapy in patients with carbapenem-resistant enterobacteriaceae infections: The TANGO II Randomized Clinical Trial. Infect Dis Ther.

[26] Chaari A, Mnif B, Chtara K (2015). Efficacy of tigecycline-colistin combination in the treatment of carbapenem-resistant Klebsiella pneumoniae endocarditis. J Glob Antimicrob Resist.

[27] Garrouste-Orgeas M, Marie O, Rouveau M (1996). Secondary carriage with multi-resistant Acinetobacter baumannii and Klebsiella pneumoniae in an adult ICU population: relationship with nosocomial infections and mortality. J Hosp Infect.

[28] Ahmad S, Al-Juaid NF, Alenzi FQ (2009). Prevalence, antibiotic susceptibility pattern and production of extended-spectrum beta-lactamases amongst clinical isolates of Klebsiella pneumoniae at Armed Forces Hospital in Saudi Arabia. J Coll Physicians Surg Pak.

[29] El Salabi A, Walsh TR, Chouchani C (2013). Extended spectrum beta-lactamases, carbapenemases and mobile genetic elements responsible for antibiotics resistance in Gram-negative bacteria. Crit Rev Microbiol.

